# Supplementary material for: Novel Bifunctional Affibody Molecules with Specific Binding to Both EBV LMP1 and LMP2 for Targeted Therapy of Nasopharyngeal Carcinoma
Source: Int J Mol Sci. 2023 Jun 14;24(12):10126. doi: 10.3390/ijms241210126 (PMC10299379; doi:10.3390/ijms241210126)
Supplement: Supplementary file 1 [file ijms-24-10126-s001.zip › ijms-2393902-supplementary.pdf]

Supplementary materials

Journal:

International Journal of Molecular Science

Title:

Novel Bifunctional affibody molecules specific binding to both EBV LMP1and LMP2 for targeted therapy of nasopharyngeal carcinoma

Authors:

Saidu Kamara*, Yanru Guo*, He Wen, Ying Liu, Lei Liu, Maolin Zheng, Jing Zhang, Luqi Zhou, Jun Chen, Shanli Zhu, Lifang Zhang

* These authors contributed equally to this work.

Institute of Molecular Virology and Immunology, Department of Microbiology and Immunology, School of Basic Medical Sciences, Wenzhou Medical University, Wenzhou 325035, Zhejiang, PR China.

Correspondence: Lifang Zhang (wenzhouzlf@126.com), +8613634286323

Supplementary Table S1. Primary antibodies used for Western blotting assay

Antibody	Catalog number	Source
P-Raf-1$^{(Ser338)}$	9427S	Cell Signaling Technology
Raf-1	53745S	Cell Signaling Technology
p-MEK1/2$^{(Ser217/Ser221)}$	9154S	Cell Signaling Technology
MEK1/2	4694S	Cell Signaling Technology
p-ERK1/2$^{(Thr202/Thr204)}$	4370S	Cell Signaling Technology
ERK1/2	4695S	Cell Signaling Technology
p-P90RSK$^{(Ser380)}$	11989S	Cell Signaling Technology
p90RSK	9355S	Cell Signaling Technology

c-Fos	2250S	Cell Signaling Technology
c-Myc	18583S	Cell Signaling Technology
GAPDH	AB-M-M001	Hangzhou Goodhere

Supplementary Figure S1. The binding ability of different concentrations of purified affibody molecules to LMP1 **A-E** and LMP2 **F-J** using SPR. **E and J**. Z_{WT} set as affibody negative control.

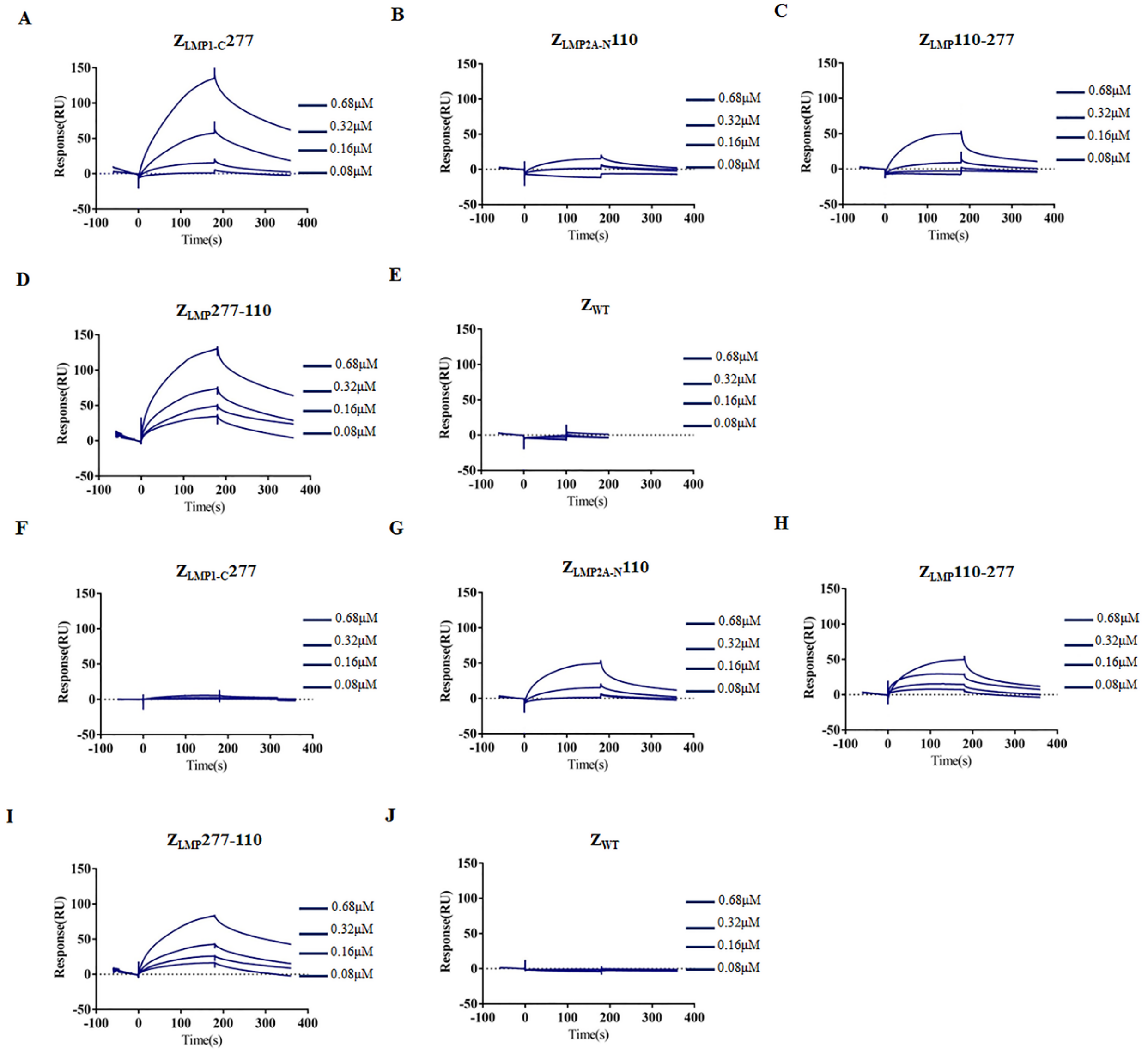

Supplementary Fig. S2. The fluorescence intensity of C666-1 incubated with Z_{LMP}277-110 was higher than that incubated with Z_{LMP}110-277.

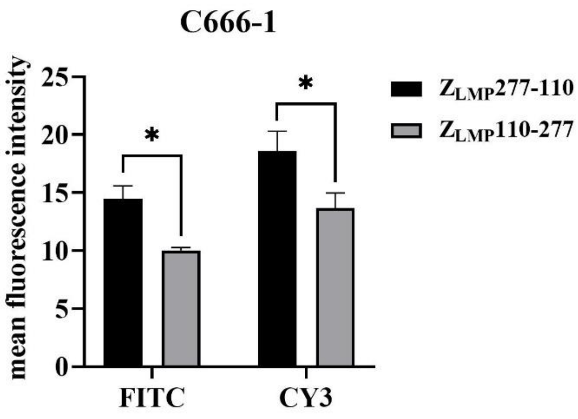

Fig. 1. Mean fluorescence intensity of C666-l cell treated with Z_{LMP}277-110 and Z_{LMP}110-277

Supplementary Figure S3. In vivo biodistribution of affibody molecules in healthy nude mice. **A and B.** After tail vein injection with Dylight 755-labelled affibody molecules, fluorescence images were obtained from mice at different time points. Kidney uptake was prominent for the accumulation of affibody molecules. The accumulation of affibody molecules maximally occurred at 8 h after injection and then decreased over the time course. The signal was undetectable at 72 h.

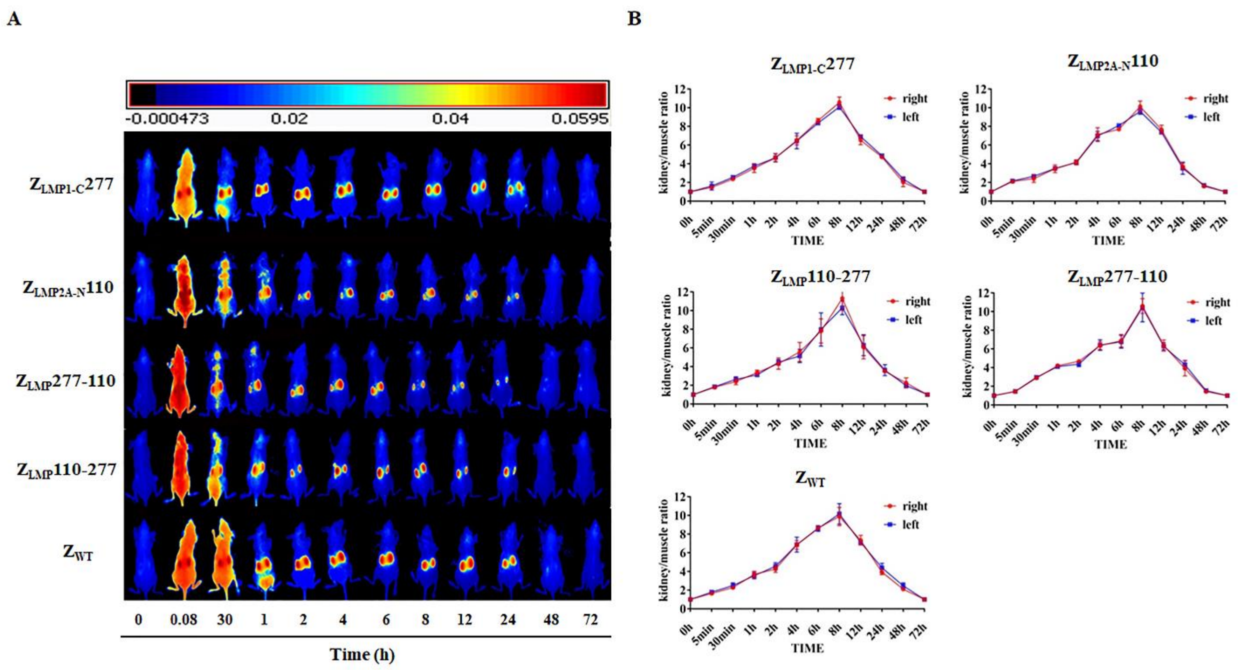

Supplementary Figure S4. **A-D** C666-1 and **E-H** CNE-2Z cells were treated with different concentrations of Z_{LMP1-C} affibody for 72 h. IC50 values were calculated using graph pad prism.

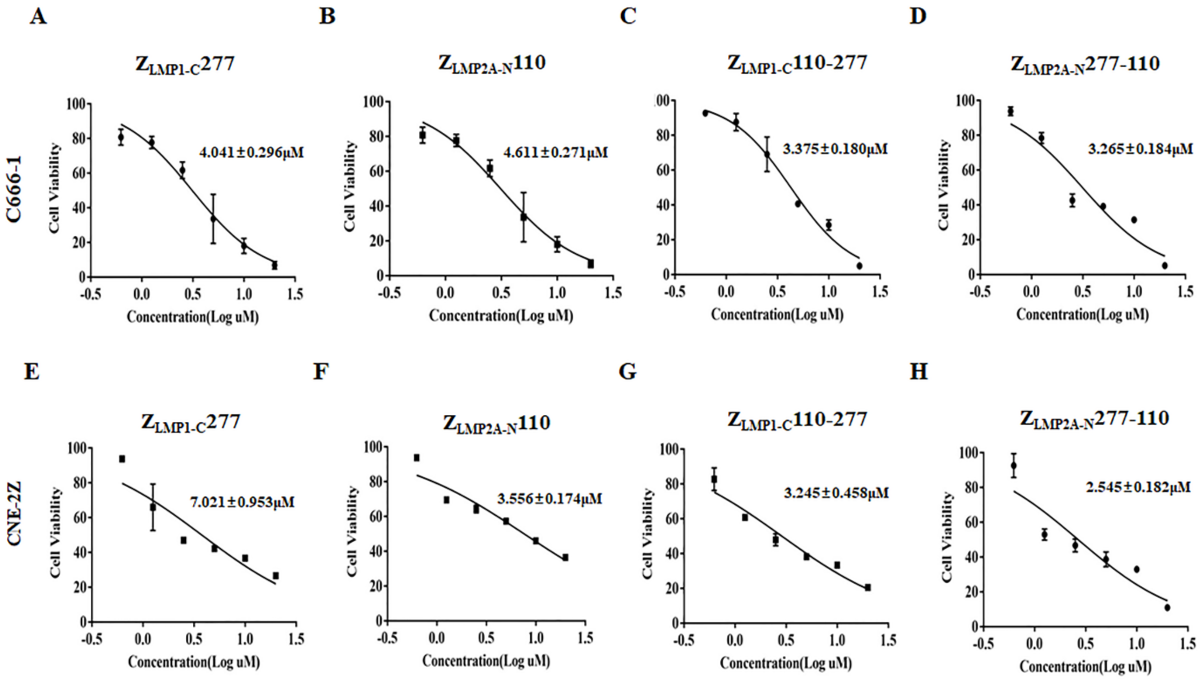

A
Z_{LMP1-C}277
C666-1
Cell Viability
Concentration(Log uM)
4.041±0.296μM
B
$Z_{LMP2A-N}$110
4.611±0.271μM
C
Z_{LMP1-C}110-277
3.375±0.180μM
D
$Z_{LMP2A-N}$277-110
3.265±0.184μM
E
Z_{LMP1-C}277
CNE-2Z
7.021±0.953μM
F
$Z_{LMP2A-N}$110
3.556±0.174μM
G
Z_{LMP1-C}110-277
3.245±0.458μM
H
$Z_{LMP2A-N}$277-110
2.545±0.182μM
